# Agent-Based Simulation for Infectious Disease Modelling over a Period of Multiple Days, with Application to an Airport Scenario

**DOI:** 10.3390/ijerph20010545

**Published:** 2022-12-29

**Authors:** Thomas Harweg, Mathias Wagner, Frank Weichert

**Affiliations:** 1 Department of Computer Science, TU Dortmund University, Otto-Hahn-Str. 16, 44227 Dortmund, North Rhine-Westphalia, Germany; 2 Department of Pathology, University of Saarland Medical School, Homburg Saar Campus, Kirrberger Strasse 100, 66424 Homburg Saar, Saarland, Germany

**Keywords:** COVID-19, agent-based simulation, social-force model, numerical simulation, systems biology

## Abstract

With the COVID-19 pandemic, the role of infectious disease spreading in public places has been brought into focus more than ever. Places that are of particular interest regarding the spread of infectious diseases are international airport terminals, not only for the protection of staff and ground crew members but also to help minimize the risk of the spread of infectious entities such as COVID-19 around the globe. Computational modelling and simulation can help in understanding and predicting the spreading of infectious diseases in any such scenario. In this paper, we propose a model, which combines a simulation of high geometric detail regarding virus spreading with an account of the temporal progress of infection dynamics. We, thus, introduce an agent-based social force model for tracking the spread of infectious diseases by modelling aerosol traces and concentration of virus load in the air. We complement this agent-based model to have consistency over a period of several days. We then apply this model to investigate simulations in a realistic airport setting with multiple virus variants of varying contagiousness. According to our experiments, a virus variant has to be at least twelve times more contagious than the respective control to result in a level of infection of more than 30%. Combinations of agent-based models with temporal components can be valuable tools in an attempt to assess the risk of infection attributable to a particular virus and its variants.

## 1. Introduction

Numerous international airports worldwide allow passengers and visitors to find a variety of restaurants and food outlets, and passengers with a valid boarding pass can also make purchases from a range of items available at duty-free stores inside the security area [1]. Aviation industry ground crew members and franchise staff are, therefore, engaged in various roles at airports, such as operating the information desks, check-in desks, and boarding desks, the security checkpoints, shops, and restaurants. Despite the fact that they constitute the main set of airport personnel that passengers and visitors will interact with at an international airport directly, their role in the COVID-19 pandemic has not been subjected to extensive simulation studies so far. We, therefore, propose a novel agent-based approach to infectious disease modelling, which we extend into covering a greater time span by simulating multiple consecutive days. We then apply this approach to a realistic scenario, modelling an international airport terminal. In this scenario, we simulate the upcoming of a new and more contagious virus variant with a gradually increasing proportion in the population with respect to an already prevailing virus variant. This approach is based on the fact that COVID-19 is linked to an enveloped, positive-sense single-stranded RNA ((+)ssRNA) virus that has been isolated from bronchoalveolar lavage fluid samples obtained from patients who contracted pneumonia in Wuhan, the People’s Republic of China (syn.: PRC) [2]. The earliest case of an infection attributable to this virus seems to have been found on 17 November 2019 [3]. On 11 February 2020, the International Committee on Taxonomy of Viruses (ICTV) announced “Severe Acute Respiratory Syndrome CoronaVirus 2” (syn.: SARS-CoV-2) as the name of the virus, formerly known as “2019 novel coronavirus” (syn.: 2019-nCov). The same day, the disease name has been designated as “CoronaVirus Disease 2019” (syn.: COVID-19) by the World Health Organization (WHO).

We propose a novel agent-based approach to infectious disease modelling, which we extend into covering a greater time span by simulating multiple consecutive days. Our approach rests upon a numerical simulation of a social force pedestrian model [4,5], which we complement with infectious disease modelling. We then apply this approach to the aforementioned realistic scenario, modelling an airport terminal. In this scenario, we simulate the upcoming of a new and more contagious virus variant with a gradually increasing proportion in the population with respect to an already prevailing virus variant.

A variety of approaches to infectious disease spread modelling have been implemented so far. They largely differ in granularity and the aspects modeled. Very common are compartmental models, which model infection dynamics on a large scale. There are several variants, notably SEIR (susceptible–exposed–infectious–recovered) and SIR (susceptible–infected–removed) and other similar models [6]. In these approaches, the population is divided into several categories, which form the basis of a system of differential equations to model infection dynamics.

On a smaller scale, various agent-based models (ABM), also called individual based models (IBM), to infectious disease modelling have been proposed [7], many of which are graph-based at their core, combined with compartmental models.

More recently, “microscopic” agent-based models have been proposed (e.g., [5,8,9,10]), which employ particle simulations, which are governed by social-force interaction, allowing for analysis of disease spreading on a small scale. While these approaches allow for highly detailed analysis in the spatial domain, they usually lack the temporal aspects of the macroscopic approaches.

We attempt to narrow this gap by extending the social force approach into covering a longer timespan. Our goal is to retain the geometric detail of ABM-based simulations, but at the same time, stretch this approach towards time spans that are relevant to covering infections caused by individuals who themselves became infected during the simulation. In other words, we seek to investigate the effect of agents passing the virus on to other agents. Due to the fine-grained nature of ABM, a simple increase in simulation time to cover days or even weeks would demand very high resources, mainly in terms of computational power, but also in modelling the underlying scenario. To overcome this issue, we propose an approach, in which we simulate a certain time span of a whole day, but repeatedly over several consecutive days. To create consistency between consecutive days, we keep a subset of the simulated agents and their state persistent over the whole set of days. These agents can be thought as, for example, employees, staff, or other people who have a regular schedule of appearance on the simulated scenario.

**Figure 1:**
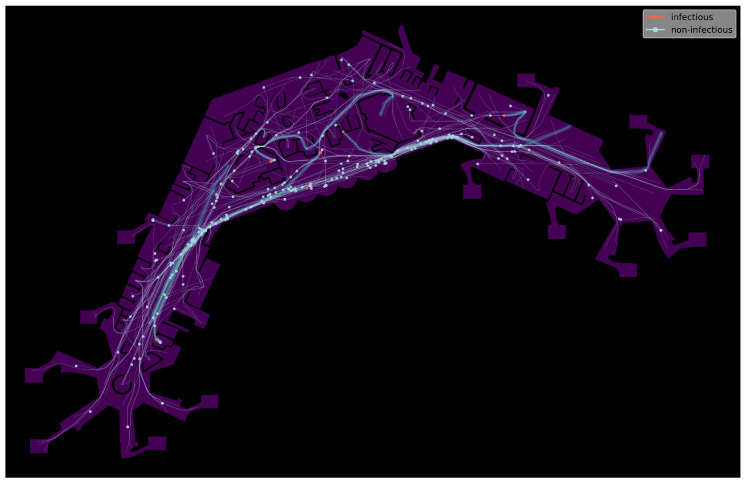
Visualisation of the developed agent-based simulation with agents, trajectories, and tracking of aerosols shown.

We then apply this approach to the analysis of infectious disease spreading by the example of two (hypothetical) variants of the SARS-CoV-2-virus with different degrees of infectivity, taking place in a realistic scenario set in an international airport terminal. With the passage of time, the emergence of more transmissible variants cannot be ruled out [11]. We provide experiments to gain insight into how high the proportion of such a variant has to be for a significant rise in infection counts. We also quantify the rate of contagiousness by quantifying the viral load necessary for infection. We include a simple model for tracking aerosol concentration and measuring aerosol exposure times in multiple discrete simulations, which are set to take place on several consecutive days. This way, we seek to combine the microscopic social-force-based simulation with the progression of infections after initial exposure. A visualisation of the simulation process is shown in Figure 1.

The main contributions of this paper are centred around a generic agent-based model for infectious disease modeling, which is:

- almost arbitrarily applicable to scenarios defined by a two-dimensional floor-plan
- capable of handling large areas and high agent counts
- incorporates a basic model of aerosol spreading
- augmented by extending the time span over multiple days to account for the temporal progress of infection dynamics

This paper is organized as follows: The following Section 2 gives an overview of the state-of-the-art of infectious disease modeling, with a focus on agent-based simulations. In Section 3, we describe in detail the model we developed and the accompanying implementation. This section is further divided into sub-sections covering pedestrian dynamics (Section 3.1), modelling of the simulation domain, including agent pathfinding (Section 3.2), and infectious disease modeling (Section 3.4). Section 4 describes our experimental setup, in Section 5, the results are presented, which are then discussed in Section 6. Finally, Section 7 gives a short conclusion and outlook.

## 2. Related Work

In this section, we first review extensions that have been made to compartmental models for covering spatial aspects, indoor disease transmission, and multi-strain epidemics, before we put our focus on agent-based models for infectious disease and epidemics modeling. We differentiate these approaches into two categories, compartmental agent-based models and social-force agent models.

Transmission of COVID-19 and airborne diseases, in general, has already been investigated with respect to indoor environments. Both Gao et al. [12] and Noakes et al. [13] use the Wells-Riley equation [14,15] in combination with an SEIR-model to investigate the effect of ventilation control and further measures on infection dynamics.

There have also been numerous approaches for analysing multi-strain pandemics with compartmental models. Lazebnik and Bunimovich-Mendrazitsky propose a model for multi-strain pandemics with a focus on the relations of basic reproduction number, the total count of infected individuals, and mortality rate [16]. In another publication, Lazebnik et al. [17] present a graph-based spatio-temporal model based on a SIIRD-scheme (susceptible–infected asymptomatic–infected symptomatic–recovered–dead) for analysis of COVID-19-related dynamics. Fudolig and Howard [18] analyse a modified SIR model, which takes vaccination into account, hence described as SVIR (susceptible–vaccinated–infected–removed) and analyse its dynamics regarding local stability. Khyar and Karam [19] follow a similar approach, analysing a multi-strain SIR model with regard to global stability. Edilson et al. [20] present an analysis and accompanying optimal control strategies of a multi-strain epidemic model with respect to COVID-19, and Yagan et al. [21] use a model proposed by Eletreby et al. [22] to analyse the impacts of an upcoming, more transmissible strain of COVID-19. De León et al. propose a multi-strain model, which they apply to the analysis of two virus variants and associated diminished effectiveness of vaccination [23].

Concerning compartmental agent-based models, Hoertel et al. propose a stochastic model, which they use for a simulation based on data from France; study the impact of measures like physical distancing, mask-wearing, and shielding individuals [24]. Their model is based on the model of Perez and Dragicevic [25], which is an SEIR-based model based on GIS (Geographic Information System) data and movement rules, and the model Venkatramanan et al. [26], which is an agent-based network model combined with SEIR, used for forecasting an Ebola outbreak. Wang et al. implemented the model of Hoertel et al. into a visual interactive tool for strategy assessment [27]. A large-scale ABM with the SIRD (susceptible–infected–recovered–deceased) scheme is proposed by Giacopelli [28]. Müller et al. combine transportation modelling with infection modelling, using “(daily) activity chains” [29]. Wolfram presents an analysis of graph-based (social-)networks and SIR-models, with studies modelling choices in general, and choice of distributions in particular [30]. Ying and O’Clery apply a graph-based model to a supermarket setting [31], and Kerr et al. propose the “Covasim” simulation framework [32], which offers various contact network models with compartmental models. It also includes a scaling model (one person represents multiple real persons) with a technique they call “dynamic rescaling”. Krivorotko et al. [33] use the Covasim-framework to consider cases of corona outbreaks in the US and UK and corresponding available data. Their focus lies on the process of data collection and parameter identification, and calibration. Truszkowska et al. propose an agent-based model with single-individual resolution and a five-state-model (susceptible–exposed–infectious-symptomatic–removed-healed–removed-dead) [34]. Shamil et al. simulate the spread of COVID-19 in the example of US cities with an agent-based state model. They also investigate the influence of the ability to trace individuals via smartphones on the accuracy of their simulation [35]. Chumachenko et al. propose an agent-based model with an SEIR scheme for assessment of the dynamics of influenza and acute respiratory virus infection [36]. Alvarez Castro and Ford combine a 3D-agent model with an SEIR model to simulate COVID-19 transmission in university students [37].

Social-force ABM has been proposed and applied to different scenarios with respect to infectious disease modelling. We already used social-force ABM for analysis of supermarket capacity or occupancy with respect to abiding prescribed distances [5]. Parisi et al. also did a study concerning supermarkets, with regard to the question of how the number of customers affects social distancing [9]. Islam et al. analysed indoor spaces in general [38]. Espitia et al. [39] propose a “social distancing model”, comparing the social force model to their own social distancing model. Their simulation considers the city of Venice. Garcia et al. analyse daily life situations in various public places [8]. Alam and Ahsanul [40] developed a social-force-based model to reflect pedestrian behaviour under COVID-19 restrictions. They also consider an airport scenario. Cuevas presents an agent-based model for COVID-19 transmission with a focus on agent interactions [41]. Nikoohemat et al. focus on building realistic point-cloud models for simulation [42], while Kramer and Wang did a study on the adverse effects of social distancing on pedestrian movement [43]. Last but not least, Mayr and Köster propose an “optimal steps model”, with focus on keeping distances [10].

In this study, we extend a social-force-based simulation [4,5] by creating coherence over multiple simulation runs, which are considered to be taking place on consecutive days. In the following sections, we describe all aspects of our simulation framework in detail, including pedestrian dynamics, modelling of the airport area, and infectious disease modelling.

## 3. Materials and Methods

In this section, we present the underlying model used in our simulation as well as the additions we made for infectious disease modelling. We first describe the pedestrian simulation itself (Section 3.1), before we proceed to infectious disease modelling (Section 3.4), including the important extension to simulating multiple consecutive days (Section 3.4.2). All identifiers and variables used throughout this chapter are summarised in Table 1.

### 3.1. Pedestrian Dynamics

**Figure 2:**
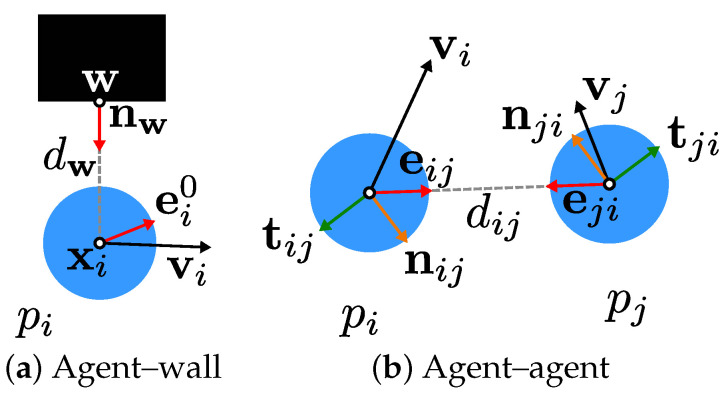
Interactions according to the underlying model of pedestrian dynamics between (**a**) agents (shown as blue points) and obstacles (black rectangle) and (**b**) between two agents, with associated quantities.

Pedestrians are modelled as self-propelled agents $p_{i}$, interacting with each other and with obstacles [4]. We formally define an agent as $p_{i}=\left(x_{i},v_{i},e_{i}^{0},v_{i}^{0},s_{i},d_{i},c_{i},c_{i}^{\mathrm{vt}},c_{i}^{\mathrm{aexp}},d_{i}^{\mathrm{inc}}\right)$, which will be explained in detail in the following. Agent interaction is effected by forces, which obstacles and other agents exert on an agent. Figure 2 shows the interaction of agents with the environment and with each other and the associated variables.

The movement of an agent is described by the following equation of motion, defining the acceleration of an agent $p_{i}$ as

(1) $$\frac{dv_{i}}{dt}=f_{i}^{0}+f_{i}^{\mathrm{wall}}+f_{ij}.$$

In this equation, $f_{i}^{0}$ denotes the self-acceleration of agent $p_{i}$, and $f_{i}^{\mathrm{wall}}$ and $f_{ij}$ denote the forces exerted by the closest obstacle, and by another agent $p_{j}$, respectively. An agent’s velocity $v_{i}$ is the derivative of its position $x_{i}$ with respect to time $v_{i}=\frac{dx_{i}}{dt}$. Note that agents have no defined mass in the underlying model, which hence is omitted. The self-acceleration $f_{i}^{0}$ is defined as

(2) $$f_{i}^{0}=\frac{v_{i}^{0}e_{i}^{0}-v_{i}\left(t\right)}{\tau },$$

where $v_{i}^{0}$ is the desired speed of $p_{i}$ (in metres per second), $e_{i}^{0}$ its desired direction, and τ is a relaxation constant, measured in seconds.

The force $f^{\mathrm{wall}}$ exerted from an obstacle $w\in R^{2}$ on agent $p_{i}$ is given by

(3) $$f_{i}^{\mathrm{wall}}\left(d_{w}\right)=a\;\exp\left(\frac{-d_{w}}{b}\right)n_{w},$$

with a=3,b=0.1 being constants [4], $n_{w}$ being the normal vector pointing from obstacle w to $p_{i}$, and $d_{w}$ the distance from $p_{i}$ to w. A detailed definition of obstacles and modelling of the simulation domain is given in Section 3.2.

The force $f_{ij}$ acting from agent $p_{j}$ on agent $p_{i}$ is defined as

(4) $$f_{ij}\left(d,\theta \right)=-\hat{a}\exp\left(\frac{-d}{\hat{b}}\right)\left[\exp\left(-{\left(n^{\prime }\hat{b}\theta \right)}^{2}\right)t_{ij}+\hat{k}\exp\left(-{\left(n\hat{b}\theta \right)}^{2}\right)n_{ij}\right].$$

Here, $e_{ij}$ denotes the normalised direction from $p_{i}$ to $p_{j}$: $e_{ij}=\frac{x_{j}-x_{i}}{∥x_{j}-x_{i}∥}$, $t_{ij}=\frac{t_{ij}^{\prime }}{∥t_{ij}^{\prime }∥}$ is the so-called interaction direction between two agents, with $t_{ij}^{\prime }=\lambda \left(v_{i}-v_{j}\right)+e_{ij}$, λ a constant, and $n_{ij}$ the normalised vector perpendicular to $t_{ij}$, oriented to the left. The angle between $t_{ij}$ (interaction direction) and $e_{ij}$ (vector pointing from $p_{i}$ to $p_{j}$) is described by $\theta_{ij}$, and $a,\hat{b},n,n^{\prime }$ are (constant) model parameters [4]. Finally, $\hat{b}$ and $\hat{k}$ are calculated as $\hat{b}=\gamma ∥t^{\prime }∥$ and $\hat{k}=\mathrm{sgn}\left(\theta \right)$.

### 3.2. Simulation Domain Modelling and Pathfinding

In this paper, we focus our simulation domain solely on the airport-terminal scenario described in Section 4 and the according floor-plan. Nonetheless, our approach is not restricted to a certain type of scenario at all, and should be applicable to almost any 2D scene described by a suitable binary floor plan.

The simulation domain is defined as a two-dimensional rectangular area $\Omega =\left[0,w\right)\times \left[0,h\right)\subset R^{2}$ of a given size w×h (in metres). The floor-plan is modelled as a two-dimensional binary map $F\in {\left\{0,1\right\}}^{w_{F}\times h_{F}}$, defining walkable space (F=1) and obstacles (F=0), the ratio $s_{F}=\frac{w_{F}}{w}=\frac{h_{F}}{h}$ defines the resolution of the floor map with respect to the underlying area in points per metre. The corresponding mapping from the simulation domain is referred to as the scalar field $\phi^{F}\left(x\right):\Omega \to \left\{0,1\right\}$. The set of obstacles can, thus, be defined as $W=\{w\in \Omega |\phi^{F}\left(w\right)=0\}$. Within Ω, the distance $d_{w}$ (cf. Equation (3)) is calculated as the perpendicular distance from $p_{i}$ to the closest obstacle w∈W.

This is done efficiently by means of the distance transform [44,45] of the binary floor map F, which is described as a scalar field $\phi^{\mathrm{dt}}:\Omega \to R$. Formally, this is defined as $\phi^{\mathrm{dt}}\left(x\right)=\min_{w\in W}\left(∥x-w∥\right)$ with $W=\{w\in \Omega |\phi^{F}\left(w\right)=0\}$.

On the area Ω, we define a set *S* of starting points $s_{k}\in \Omega \subset R^{2}$ and a set *D* of destination points $d_{k}\in \Omega \subset R^{2}$, which determine how the agents move and navigate in the simulation. The set of destination points *D* is further divided into two disjoint sets, $D=D_{D}\dot{\cup }D_{W}$, denoting waypoints ($D_{W}$) and final destination points ($D_{D}$). Technically, both are mostly treated the same way, the only difference arises in handling agents after they reach those points. This is described in detail in the following. Regardless of the type of destination point, agents start from a certain point $s_{i}$ from the set *S* and move towards a point $d_{i}\in D$. Within the simulated airport terminal, starting points represent entrances or incoming escalators within the simulated airport. Waypoints represent points-of-interest of any kind agents by stopping by, like counters, helpdesks, or shops, while final destination points represent exits or outgoing escalators.

**Figure 3:**
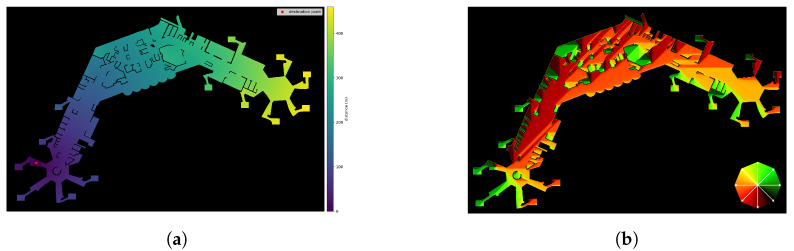
Exemplary visualisation of distance map $\phi^{\mathrm{dist}}$ and directions $G^{\mathrm{dir}}$, the associated destination point is located in the lower left in both cases. (**a**) Colour-coded display of a distance map $\phi^{\mathrm{dist}}$. (**b**) Colour-coded display of directions $G^{\mathrm{dir}}$, mapping of directions to colours is shown in the lower right.

Path-finding is done by applying Dijkstra’s Algorithm [46] to the floor plan, generally assuming an 8-neighbourhood-connectivity at each point x∈Ω of the floor-plan F. Depending on the problem, other algorithms can also be integrated for the pathfinding, like, for example, A* [47] or fast-marching based methods [48,49]. This results in scalar fields $\phi_{k}^{\mathrm{dist}}:\Omega \to R$, one for each way- and destination point $d_{k}\in D$. Each of those maps consequently holds the minimum distance to the destination point for each walkable position $\phi^{F}=1$. Following, the gradient operator is applied to the distance field giving a vector field $G_{k}^{\mathrm{dir}}=\nabla \phi_{k}^{\mathrm{dist}}$, $G_{k}^{\mathrm{dir}}:\Omega \to R^{2}$. Exemplary colour-coded visualisations of the resulting scalar- and vector fields are shown in Figure 3a and Figure 3b, respectively. The colours in Figure 3a indicate the distance to the destination point, from near (violet) to far (yellow). In Figure 3b, the colours correspond to directions of the shortest path at every position, as shown in the colour-wheel at the lower right. For example, pure orange colour implies movement to the left, and pure green colour implies the upper right direction. Blended colours accordingly stand for directions in between.

Agents have several attributes attached beside the model-related value of desired speed $v_{i}^{0}$, including the current starting point $s_{i}\left(t\right)\in S$ from the set of available points, and the current destination point $d_{i}\left(t\right)\in D$. Additional attributes relevant to infectious disease modelling will be detailed in Section 3.4.

### 3.3. Pedestrian Simulation

**Figure 4:**
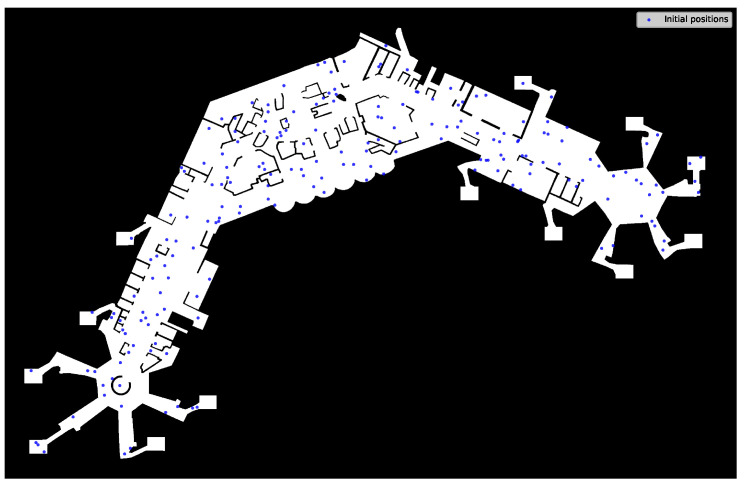
Exemplary starting point configuration, individual starting positions are shown as blue points.

The simulation runs on a defined set of agents $P_{\mathrm{sim}}$ of given size $n_{P}=\left|P_{\mathrm{sim}}\right|$ at the same time, and over a defined period of time $\left[t_{0},t_{\max}\right)$, measured in seconds, with $t_{0}=0$ by default. Initially, each agent is assigned a destination point $d_{i}\left(t_{0}\right)$, which is chosen uniformly random from the set *D* of available points. However, to avoid crowding around starting points at the beginning of the simulation, which would also lead to distorted data concerning aerosol exposure levels (cf. Section 3.4.1), the initial starting positions $s_{i}\left(t_{0}\right)$ of the agents are spread randomly across the (walkable) area of the terrain, instead of drawing from the available starting points *S*. These points are used only afterward, as described below. A possible starting point configuration is shown in Figure 4.

Even though the number of agents in the simulation is set to a fixed number $n_{P}$, as stated above, the number of individuals in a simulation run can be notably higher by allowing for individuals to leave and new individuals to join. To this end, a global agent pool $P_{\mathrm{pool}}$, with $|P_{\mathrm{pool}}\left|>\right|P_{\mathrm{sim}}|$, is maintained. This pool defines the individuals available for the simulation in its entirety. If, after reaching its destination, an agent leaves the simulation, it gets replaced by another individual from the agent pool. The new agent is then randomly assigned a starting point $s_{i}\left(t\right)\in S$ and a way- or destination point $d_{i}\left(t\right)\in D$. However, if an agent’s current destination $d_{i}$ is a waypoint, i.e., $d_{i}\in D_{W}$, this agent is not removed from the simulation after it reaches this point. Instead, it is randomly assigned a new destination $d_{i}$ from the set *D*. This means, the new destination can again be either a way- or a destination point, and the process repeats until the agent reaches a final destination point from $D_{D}$. As the computation of inter-agent forces have to be computed for all pairs implying a complexity of $O(n^{2})$, running the simulation is quite computationally intensive. Thus, for efficient execution, we devised parallel implementations for both conventional desktop processors (CPU, Central Processing Unit) and dedicated graphics processors (GPU, Graphics Processing Unit), using OpenMP (https://www.openmp.org/, accessed on 23 September 2022) and OpenCL (https://www.khronos.org/opencl/, accessed on 23 September 2022), respectively. Our analyses revealed that the OpenMP-based implementation did not yield a major improvement in our experiments, while the OpenCL implementation did, with a speed-up by a factor of almost 10.

In the following section, we describe the additions we made to the pedestrian model for modelling infectious disease transmission.

### 3.4. Infectious Disease Modelling

We model virus transmission by generating simulated aerosol trails based on the agents’ trajectories. The resulting viral loads are then accumulated over time and a rough approximation to diffusion is applied. To evaluate a simulation run with regard to different infection scenarios, aerosol trail generation is not done while the simulation is running, but rather afterwards.

Specific to infectious disease modelling, the following attributes are attached to an agent $p_{i}$: Its infectivity $c_{i}\in \left\{0,1\right\}$ as a Boolean value, the according virus variant $c_{i}^{vt}\in \left\{0,1\right\}$, if $c_{i}$ is true (i.e., 1), and the cumulative viral load from aerosol exposure time $c_{i}^{\mathrm{aexp}}$.

#### 3.4.1. Aerosol Modelling

**Figure 5:**
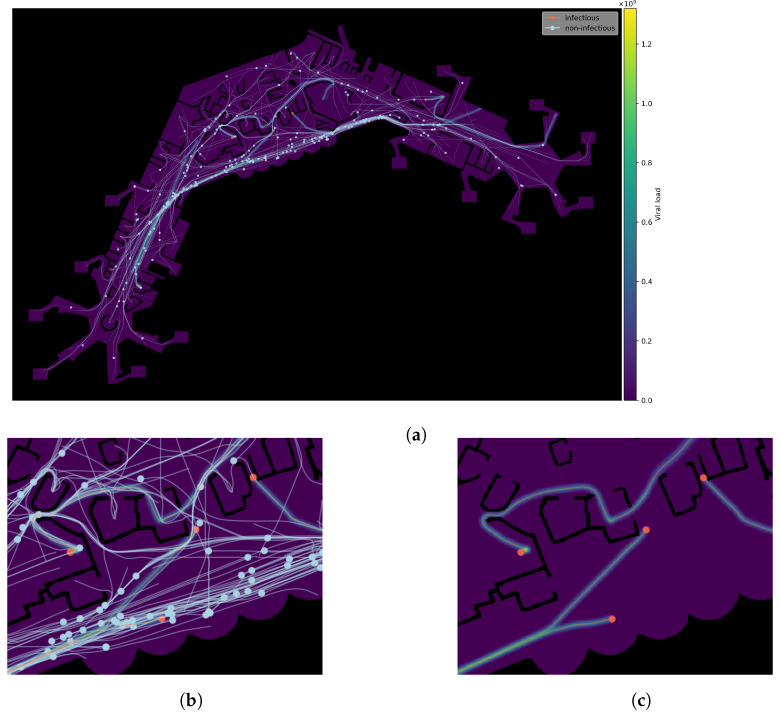
Detail view of trajectories and aerosol trail generation. (**a**) Exemplary agent trajectories and aerosol trail generation. (**b**) Detail view with agents, trajectories, and aerosol traces. (**c**) Detail view showing only infectious agents and traces.

As already mentioned, virus transmission is modelled by tracking aerosol concentration within the simulated area. To this end, we augment our simulation with a simple aerosol model we developed specifically for this purpose. The calculation is done as follows. The movement data containing the agents’ positions $x_{i}\left(t\right)$ for $t\in [t_{0},t_{\max})$ is split into discrete time-frames of length $t_{\mathrm{frame}}$ (measured in seconds) and sampled at a rate of $t_{\mathrm{steps}}$ steps per frame. Aerosol maps, represented as scalar fields $\phi_{j}^{ae}:\Omega \to R$, are then generated as follows, one for each time frame. For each (sampled) position of an infected agent’s $p_{i}$ trajectory, a certain amount $c^{\mathrm{ae}}$ of virus concentration is distributed evenly and proportionally to the area of a disc of radius $r_{\mathrm{ae}}$ (in metres), centred at an agent’s current position $x_{i}\left(t\right)$ for $t\in [t_{j},t_{j+1})$ with $t_{0}=0$ and $t_{j+1}=t_{j}+t_{\mathrm{frame}}$. Thus, each map $\phi_{j}^{ae}$ covers a time-window of length $t_{\mathrm{frame}}$ seconds. Virus concentration is accumulated not only within a map $\phi_{j}^{ae}$ of a single discrete time frame but over consecutive maps $\phi_{j}$ and $\phi_{j+1}$, as well. Additionally, aerosol diffusion is approximated by applying a Gaussian filter (with parameter σ, relative to map resolution) to the aerosol map within each step, after all trails have been calculated. To inhibit diffusion through walls, all areas which represent obstacles are then set to zero. A visualisation of the process can be seen in Figure 5, with Figure 5a showing an aerosol map, with agents and their trajectories as overlays. Infectious agents are drawn as red discs and non-infectious ones in light blue. Trajectories are shown as lines in the same colours. For better recognisability, a section from the centre is shown again in greater detail, with Figure 5b showing all agents and trajectories and Figure 5c showing only infectious ones, which are the ones responsible for generating aerosol-trails with viral load.

**Figure 6:**
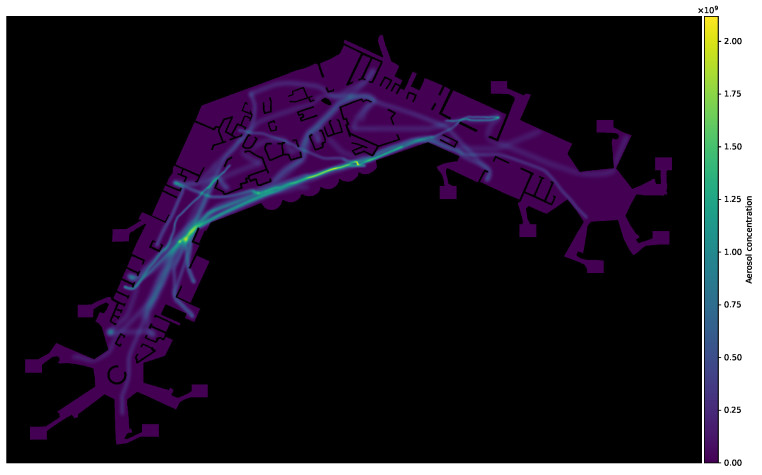
Exemplary visualisation of aerosol trails and corresponding amount of viral load.

Tidal air virus concentration is measured in $\mathrm{mRNA}\;\mathrm{copies}\;\;\cdot \;{\mathrm{cm}}^{-3}$ [50], the actual value $c_{i}^{\mathrm{ae}}\left(t\right)$ is sampled from a normal distribution at each step. The actual virus concentration deposited is calculated as the product of respiration rate $r_{\mathrm{resp}}$, viral load $c_{i}^{\mathrm{ae}}\left(t\right)$, and length of the time-step $t_{\mathrm{steps}}$. Figure 6 shows an exemplary visualisation of aerosol concentration level within the simulation area.

Finally, aerosol exposure measurement is done analogously to the generation of aerosol trails. For each step *j*, aerosol concentration, given by the corresponding aerosol map $\phi_{j}^{\mathrm{ae}}$, is integrated over the (non-infected) agents’ trajectories as the (line-)integral along the section of the trajectory of agent $p_{i}$ over the aerosol-map $\phi^{ae}$. Thus, aerosol concentration for step *j* is calculated as a discrete approximation of:

(5) $$c_{i,t_{j}}^{\mathrm{aexp}}=\int_{t_{j}}^{t_{j+1}}\phi_{j}^{aec}\left(x_{i}\left(t\right)\right)dt.$$

From these series of values, the total viral load exposure per agent is calculated as the cumulative sum

(6) $$c_{i}^{\mathrm{aexp}}=\sum_{j}c_{i,t_{j}}^{\mathrm{ae}}.$$

These values are then used for determining if an agent is possibly infected, by comparing $c_{i}^{\mathrm{aexp}}$ to the threshold $c_{\mathrm{vt}}^{\mathrm{crit}}$ for the corresponding virus variant.

#### 3.4.2. Extension and Coherence over Multiple Days

The strength of our social-force-based simulation lies in tracking contact times with great geometric detail, mostly in a confined space, but, more importantly, in a rather short period of time. To overcome this limitation, we propose the following additions, to simulate coherence over time between multiple runs of the simulation. This way, not only the effects of an agent passing the infection on to other agents can be taken into consideration, but possible re-infection as well. Central to our method is a subset of agents which persists over the multiple runs of the simulation. To this end, a subset $P_{\mathrm{persist}}\subset P_{\mathrm{pool}}^{0}$ is chosen from the set of all agents available in the pool $P_{\mathrm{pool}}^{0}$ of the first simulation run. In the context of the airport terminal scenario chosen here, persistent agents would most likely correspond to personnel working at the airport. Then, a series of a total of $m_{\mathrm{total}}=10$ discrete simulations are run, each simulation representing a consecutive day in the simulated (airport terminal) setting. Each simulation is run with the same basic settings. The agent pool $P_{\mathrm{pool}}^{m}$ for each simulation following the first one are sampled randomly from the same distributions except for the set of persistent agents. From simulation-day, *m* to m+1, the set $P_{\mathrm{persist}}$ of agents are transferred to m+1 with their state being the one of the ends of the simulation of day *m*. In the transition from day *m* to day m+1, it is checked whether a persistent agents’ $p_{i}^{\mathrm{pst}}$ aerosol viral exposure level $c_{i}^{\mathrm{aexp}}$ is above a given threshold $c_{\mathrm{vt}}^{\mathrm{crit}}$ for the corresponding virus type. If this is the case, the agent is assumed to be infected by the end of a certain incubation period of $d_{i}^{\mathrm{inc}}\in R$ days, which is sampled from N(3,0.5) (cf. [51,52,53]). If this incubation period is over, the agents infection status $c_{i}$ is set to 1 (along with the according virus-type $c_{i}^{\mathrm{vt}}$). This way, the detailed spatial simulation of virus spreading is extended into the time domain.

**Table 1:** List of identifiers and parameters used.

Name	Description
*a*	Model parameter
$\hat{a}$	Model parameter (*A* in [4])
*b*	Model parameter
$\hat{b}$	Model parameter (*B* in [4])
$c_{i}$	Infectivity of $p_{i}$
$c_{i}^{\mathrm{vt}}$	Virus-variant of $p_{i}$
$c_{i}^{\mathrm{ae}}$	Tidal air virus load of $p_{i}$
$c_{i}^{\mathrm{aexp}}$	Aerosol exposure level of $p_{i}$
$c_{vt}^{\mathrm{crit}}$	Necessary viral load for infection per variant
$d_{i}^{\mathrm{inc}}$	Incubation time for $p_{i}$
$d_{w}$	Distance to wall
$d_{i},d_{k}$	Destination point
*D*	Set of all way- and destination points
$D_{D}$	Set of all destination points
$D_{W}$	Set of all waypoints
$e_{ij}$	Direction from $p_{i}$ to $p_{j}$
$f_{i}^{0}$	Self-acceleration
$f_{ij}$	Agent-to-agent force
$f_{i}^{\mathrm{wall}}$	Wall force
F	Binary floor map
$G_{k}^{\mathrm{dir}}$	Direction map for $d_{k}$
*h*	Height of simulated area in metres
$h_{F}$	Height of floorplan in pixels
$\hat{k}$	Model parameter (*K* in [4])
$m_{\mathrm{total}}$	Number of simulated days
$n_{ij}$	Vector perpendicular to $t_{ij}$
$n_{w}$	Wall normal
*n*	Number of agents
$n_{P}$	Size of agent pool
$n^{\prime }$	Model parameter
Ω	Simulation area
$\phi^{\mathrm{ae}}$	Map of aerosol concentration
$\phi^{\mathrm{dt}}$	Distance transform of F
$\phi^{F}$	Floor-plan/obstacle map
$p_{i}$	Agent
$p_{i}^{\mathrm{pst}}$	Persistent agent
$P_{\mathrm{sim}}$	Set of simulated agents
$P_{\mathrm{pool}}$	Set of all available agents
$P_{\mathrm{persist}}$	Set of persistent agents
$r_{\mathrm{ae}}$	Radius of aerosol distribution in metres
$r_{\mathrm{resp}}$	Respiration rate
$s_{i}$	Starting point of agent $p_{i}$
*S*	Set of all starting points
$s_{k}$	Starting point
$t_{ij}$	Interaction direction between $p_{i}$ and $p_{j}$
$t_{0}$	Time of simulation start
$t_{\max}$	Time of simulation end
$t_{\mathrm{frame}}$	Length of time-frame for aerosol calculation
$t_{\mathrm{steps}}$	Sampling rate for time-frames
τ	Relaxation constant
$v_{i}$	Velocity of $p_{i}$
$v_{i}^{0}$	Desired speed of $p_{i}$
w	Wall/obstacle position
*W*	The set of all obstacle positions
*w*	Width of the simulated area in metres
$w_{F}$	Width of floor-plan in pixels
$x_{i}$	Position of $p_{i}$

## 4. Experiments

**Figure 7:**
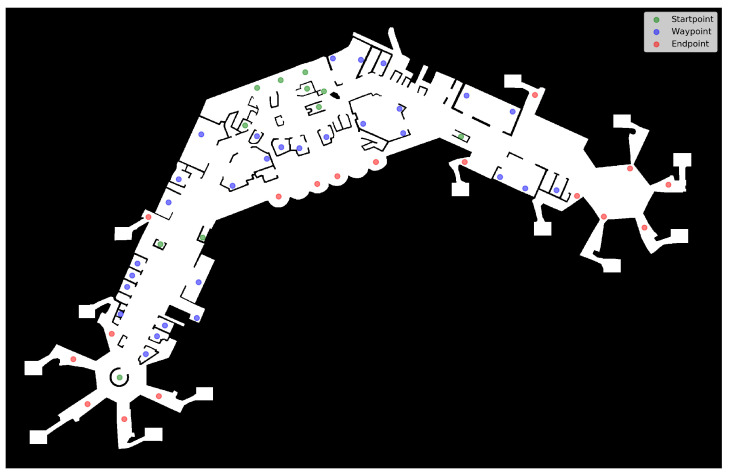
Floorplan with starting- and destination points shown.

We run the simulation in a realistic setting which represents the floorplan of an airport terminal, as shown in Figure 7. The floorplan was created true to scale, based on publicly available map data (Google Maps https://www.google.de/maps/@50.0475523,8.5731218,17.96z, accessed on 8 December 2022). The interior structure and points of interest (POI) were placed to be in accordance with the assumed layout as closely as possible. Waypoints were placed at stores and counters, starting points at assumed entries and escalators, and exit points at possible exits. The actual size is assumed to be 451.63 m × 291.78 m, the floor map $T_{F}$ has a resolution of $s_{T}=7.05$ points per metre. The size stems directly from the map data, while the resolution is resulting from the manual process of map creation. The floor map is embedded into the rectangular area Ω, in which we manually define 11 starting points (*S*) and 46 destination points (*D*). The destination points are divided into 29 waypoints ($D_{W}$) and 17 final destination points ($D_{D}$), with the numbers resulting directly from the manual placement described above. The distribution of the respective points is shown in Figure 7, as well. The considered overall time period in days is assumed as $m_{\mathrm{total}}=10$ and individual simulation time is set to $t_{\max}=3600$ (one hour). This means we simulate ten consecutive days, and for each day a time-frame of one hour. The number of days were chosen to be large enough with respect to the chosen incubation period with a mean of three days (cf. Section 3.4.2), and $t_{\max}$ was intended to be an adequate time-window per day, while keeping computing time at reasonable levels.

The number of simultaneously active agents is set to $n_{P}=\left|P_{\mathrm{sim}}\right|=200$, while the total number of individuals available from the agent pool is $|P_{\mathrm{pool}}|=3000$. Consecutive simulations from one day to the next share $|P_{\mathrm{persist}}|=50$ persistent agents. These numbers are intended to be a rough approximation to the order of magnitude of passengers at the corresponding airport with reference to the chosen time interval per day (https://www.fraport.com/en/investors/traffic-figures.html, accessed on 8 December 2022). The agents’ desired movement speeds $v_{i}^{0}$ are sampled according to N(1.29,0.19), as suggested in the original paper [4].

Time-frames for aerosol and viral load exposure calculation are set to $t_{\mathrm{frame}}=50\;s$, with a sample rate of $t_{\mathrm{steps}}=500$. These values were chosen for computational efficiency. Smaller numbers would lead to numerically more accurate results but increased computation times. The radius of aerosol distribution is chosen as $r_{\mathrm{ae}}=0.5\;m$ [54]. Tidal air virus concentration is assumed as $5\times 10^{8}\;\mathrm{mRNA}\;\mathrm{copies}\;\;\cdot \;{\mathrm{cm}}^{-3}$ [50], the actual value $c_{i}^{\mathrm{ae}}\left(t\right)$ is sampled from a normal distribution $N(5\times 10^{8},10^{3})$ each step. A person’s respiration rate $r_{\mathrm{resp}}$ is assumed as 10 L min${}^{-1}$. Finally, Gaussian blur is applied with σ=3 to approximate diffusion.

In terms of infectious disease modelling, we consider two main scenarios. In the first scenario, we only assume a single virus-variant, whereas, in the second one, infected agents carry one of two different virus-variants. For the first scenario, we only change the threshold $c^{\mathrm{crit}}$ an agent has to resorb to count as infected. For the second scenario, we additionally vary the relative amounts of the two different virus types among the infected agents.

For each simulation run of the assumed duration of ten days, the set of infected agents is divided into two different virus variants, which differ in terms of infectivity. This again is effected by defining distinct thresholds $c_{\mathrm{vt}}^{\mathrm{crit}}$ for each type, meaning the assumed viral load an individual has to resorb to get infected is significantly lower for the more infectious variant. Viral load is measured in RNA concentration, cf. [50].

We assume the amount of infected to be at 2%, so infectivity is sampled from a Bernoulli distribution: $c_{i}∼B\left(0.02\right)$. This number is an estimation of the incidence among vaccinated people at the age of 18 to 59 (https://www.rki.de/DE/Content/InfAZ/N/Neuartiges_Coronavirus/Daten/Inzidenz_Impfstatus.xlsx?__blob=publicationFile, accessed on 12 December 2022). The virus-type $c_{i}^{\mathrm{vt}}\in \left\{0,1\right\}$ is chosen from two variants, with their amounts varying in ratios 9:1, 8:2, 7:3. This again corresponds to a sampling from B(0.9) down to B(0.7).

For each agent $p_{i}$, aerosol exposure $c_{i}^{\mathrm{ae}}$ is measured as described in Section 3. An agent is considered to be infected if it’s accumulated viral load $c_{i}^{\mathrm{ae}}$ exceeds the threshold of corresponding virus type $c_{i}^{\mathrm{vt}}$. Persistent agents can cause infections of other agents–in addition to agents who have been infectious from the beginning–if they become infective within the simulated period of ten days.

We then go on to evaluate the average infection counts and how they change over the simulated duration of 10 days, for each variant of the chosen parameters. For each combination of available parameters, we perform ten separate runs of the simulation process. This is not to be confused with the discrete simulation runs for the considered period of ten days. Or, put differently, each ten-day simulation run is repeated independently ten times, to get more reliable and meaningful figures. We did not choose a higher number here, as each simulation run is relatively expensive in terms of computing time. All simulations were run on a desktop computer with an *Intel Core i9-9900K 3.6 GHz* CPU, 64 GB of RAM, and an *NVIDIA GeForce RTX 2080 Ti* GPU with 11 GB of RAM.

## 5. Results

We analyse the results of our experiments with regard to two principal questions. First, we check how different levels of infectivity affect the number of infected in our simulated scenario. This gives insight into the orders of magnitude to consider with the assumptions and parameters chosen, and furthermore ensures that our simulation behaves as expected. Second, we tackle the main question of our scenario, introducing a second, more contagious virus type, which is gradually gaining traction. Here, we are interested in the proportions of how high the ratio of the new variant is compared to the pertaining one, and how much more contagious it has to be to cause notable increase in infections.

### 5.1. Single Virus-Variant

**Figure 8:**
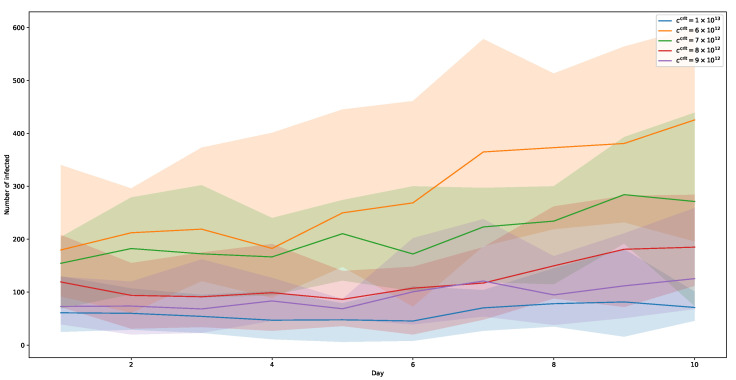
Simulations with one virus-variant of varying contagiousness: Number of infected agents over a simulated period of ten days. Shaded areas show respective minima and maxima.

We first consider the results of running the simulation with only one virus type. In different runs, we vary the contagiousness $c_{0}^{\mathrm{crit}}$ from $1\times 10^{13}$ down to $6\times 10^{12}$, one for each run. The range of these numbers was chosen based on empirical data from simulation runs in conjunction with the chosen amount of average viral load, as described in Section 3.4.1. Figure 8 shows the results of these simulations. Here, each graph shows the mean value of ten simulation runs. As to be expected, a lower threshold $c_{0}^{\mathrm{crit}}$ results in faster-growing infection rates and, thus, more infected people. With $c_{0}^{\mathrm{crit}}=1\times 10^{13}$, the curve does not rise at all until day 6, and still remains flat more or less, afterwards. Within thresholds from $0.9\times 10^{11}$ down to $0.7\times 10^{11}$, infection counts rise more obviously. And running at $c_{0}^{\mathrm{crit}}=0.6\times 10^{11}$ shows an even more significant increase in infections during the 10-day period, especially after day 5, which is in accordance with the assumed incubation time of about three days. The peak at day 10 for this threshold equates to a relative amount of about 17% of all simulated agents.

In summary, the evolution of the figures behaves as can be expected. Rising numbers due to infections by newly infected agents do not occur until about four days of the simulated overall time period. Depending on the choice of threshold $c_{0}^{\mathrm{crit}}$, and, thus, the degree of infectivity, the steepness of the curves change, with lower thresholds causing more infections.

### 5.2. Two Virus-Variants

Based on these numbers, we now consider simulations with two different virus types of different contagiousness. The first variant is kept at a constant threshold of $c_{0}^{\mathrm{crit}}=1.9\times 10^{12}$, slightly below the highest value of the single-variant runs, where the number of infections remained almost stable over the first half of the simulated period of 10 days and only a slight increase in the second half. This can be interpreted as a variant the population already has developed a certain degree of immunity, whether through vaccination of previous infections.

In separate simulation runs, we then gradually adjust the value $c_{1}^{\mathrm{crit}}$ for the second virus type. This value is chosen to be significantly lower than the one for the first virus type, $c_{0}^{\mathrm{crit}}$, as it intended to model a significantly more contagious new variant. For each value of $c_{1}^{\mathrm{crit}}$, we also ran separate simulations with the relative amounts of the infected carrying the second virus variant increasing. In other words, for each chosen value of $c_{1}^{\mathrm{crit}}$, three series of simulations are run, one for each chosen ratio of old-to-new-variant. We do this for ratios 9:1, 8:2, and 7:3. With the combination of these parameters, we seek to gain insight into the critical proportions when a new, more contagious variant gains traction.

**Figure 9:**
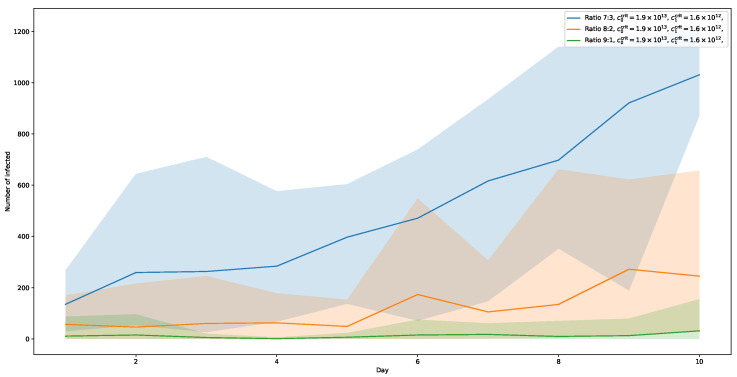
Viral load thresholds $c_{0}^{\mathrm{crit}}=1.9\times 10^{13}$ and $c_{1}^{\mathrm{crit}}=1.6\times 10^{12}$. In this setting, only the numbers for the ratio 7:3 are significantly rising.

Figure 9 shows the results of the simulation series with $c_{1}^{\mathrm{crit}}=1.6\times 10^{12}$. Here, only a ratio of 7:3 for virus types shows a significant increase in infection numbers. At its peak on day 10, a share of about 43% of the simulated population is reached. With a ratio of 8:2, the number of infected agents also rise after 5 days, but much slower. The highest relative rate (day 9) is at about 11%. Finally, with a ratio of 9:1, the curve remains almost flat, showing no noticeable effect of the more contagious virus-type added.

**Figure 10:**
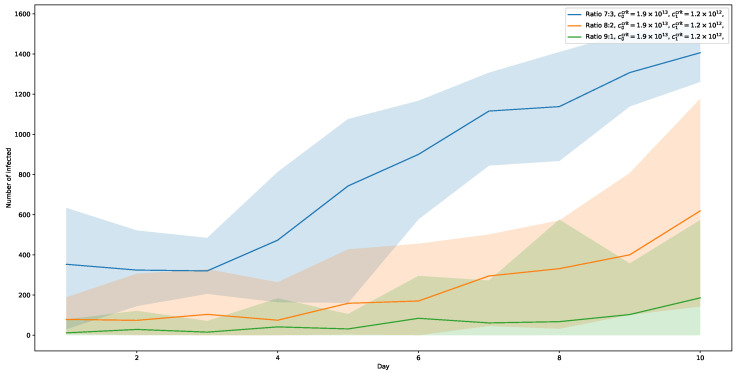
Viral load thresholds $c_{0}^{\mathrm{crit}}=1.9\times 10^{13}$ and $c_{1}^{\mathrm{crit}}1.2\times 10^{12}$. The number of infected is significantly rising for ratio 7:3, while for ratio 8:2 also rising, but much slower.

Figure 10 shows the results for $c_{1}^{\mathrm{crit}}=1.2\times 10^{12}$. In this setting, a ratio of 7:3 shows an even stronger increase (with a maximum of almost 58%), while a ratio of 8:2 also shows a notable rise in infected towards the end of the 10-day period, albeit much slower and still well below the numbers of the 7:3-runs with the higher threshold $c_{1}^{\mathrm{crit}}=1.6\times 10^{12}$. The corresponding peak is at about 26%. The runs for ratio 9:1 also start to rise slowly, but still not really noticeable.

**Figure 11:**
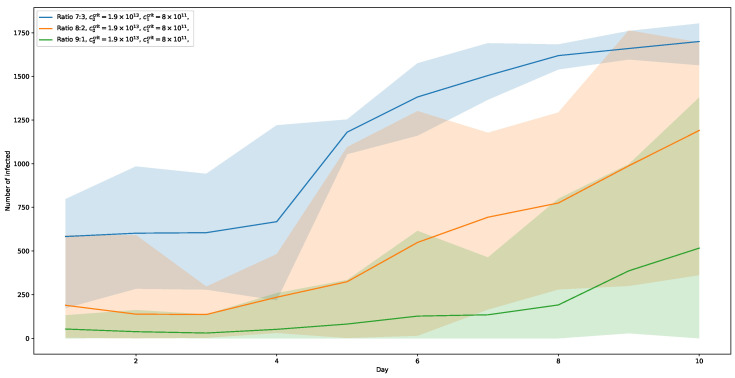
Viral load thresholds $c_{0}^{\mathrm{crit}}=1.9\times 10^{13}$ and $c_{1}^{\mathrm{crit}}=0.8\times 10^{12}$. Here, the numbers for ratio 7:3 are significantly rising with a jump after day 4. For ratio 8:2, the numbers are also significantly rising. Ratio 9:1 shows a rise towards the end of the ten-day-period.

If we further lower the threshold of the more contagious variant to $c_{1}^{\mathrm{crit}}=0.8\times 10^{12}$, the trend indicated by the previous runs continues. The corresponding curves are shown in Figure 11. Here, the 7:3-ratio shows a strong increase, with a notable jump in numbers already after day 4, ending at a share of slightly above 70%. Moreover, the curve of the 8:2-ratio-variant is much steeper than in the $c_{1}^{\mathrm{crit}}=1.2\times 10^{12}$-runs, reaching almost 50% at its peak. And even the 9:1-ratio distribution of the two virus-variants shows an increase at about day 8, still slightly above 20%, indicating that the further lowering of viral load necessary for infection shows an effect, even if the amount of infected among the population is only at 10%.

## 6. Discussion

We have presented a social-force-based simulation with a basic aerosol model for modelling the spread of infectious diseases. Central to our approach is the extension of our simulation model into covering a larger span of time (days instead of hours) by creating coherence over days. This was done by integrating a subset of individual agents for multiple runs of the simulation, which had a consistent state over the whole time period. In this process, each simulation run covers part of one of multiple consecutive days. With these assumptions made, we applied our simulation to a scenario representing an international airport terminal.

In this scenario, persistent agents can be thought of as employees, staff, or crew members. The results can provide insight into the impact of COVID-19 on the ground crew, franchise staff, or passengers at an international airport. Considering the potentially high numbers of people attending such a location and the distances potentially travelled, such a scenario could prove to be very relevant for infectious disease spread [55]. Nevertheless, as our approach is kept very generic, it can be applied to almost any scenario, which includes individuals attending on a regular short-term basis, for example, supermarkets, malls, stations, or public buildings.

As the model presented here is focused on the extension of an agent-based approach into the temporal domain, there are some limitations to our simulation. First of all, aerosol modelling is basic and could be improved by a proper fluid dynamics simulation (cf. [56,57,58,59]). Thus, accuracy is limited, and phenomena like air circulation are not taken into account yet. Both the pedestrian- and aerosol simulation are two-dimensional, even though this is presumably more relevant to the latter, as well. The agents’ behaviour is mainly governed by commuting between their points of interest, which can presumably be considered a reasonable approximation for the scenario at hand. Here, more sophisticated models of pedestrian behaviour could be incorporated [40,60]. Finally, the implemented virus- or disease-transmission model only considers the assumed viral load in the air. In this regard, a more sophisticated model of transmission (cf. [12,13]) is a possible enhancement for future research. Additional factors like an individual’s age, the wearing of various types of masks, vaccination status, or similar could also be taken into account then.

So far, the our results of our experiments appear to be plausible, with figures changing with different parameters as to be expected. Consistency with existing models, however, remains to be analysed, at least as far as the models can be directly compared. In this context, especially comparison to established temporal models of infectious disease spread should be carried out.

## 7. Conclusions

Obvious improvements can be made by incorporating a more sophisticated aerosol model based on computational fluid dynamics (CFD). Not only would this make for a more realistic account of aerosol concentration, but also aspects like ventilation could be taken into account. CFD has already been applied in various scenarios regarding the airborne spreading of diseases in general and COVID-19 in particular [56,57,58,59]. Moreover, phenomena on a smaller scale, considering droplets and local spreading [61], as well as general insight about the flow physics of COVID-19 [62] have been covered in research.

Apart from the disease modelling aspect, the agent simulation we proposed can be further improved by allowing for varying elevation levels. Extension to multiple layers or even to three dimensions might also be possible, but would take considerably more effort. Another quite obvious improvement, which could be done with little effort, could be made by not only simulating one time period per day, but rather multiple ones. A possible choice might be, for example, one period of morning hours, afternoon, and evening, respectively.

## Data Availability

Not applicable.

## Abbreviations

The following abbreviations are used in this manuscript:

ABM	Agent-Based Model
CFD	Computational Fluid Dynamics
COVID-19	Corona Virus Disease 2019
CPU	Central Processing Unit
GIS	Geographic Information System
GPU	Graphics Processing Unit
IBM	Individual-Based Model
ICTV	International Committee on Taxonomy of Viruses
mRNA	Messenger Ribonucleic Acid
OpenMP	Open Multi-Processing
OpenCL	Open Computing Language
RNA	Ribonucleic Acid
SARS-CoV-2	Severe Acute Respiratory Syndrome CoronaVirus 2
SEIR	Susceptible–Exposed–Infectious–Recovered
SIIRD	Susceptible–Infected asymptomatic–Infected symptomatic–Recovered–Dead
SIR	Susceptible–Infected–Removed
SIRD	Susceptible–Infected–Recovered–Deceased
+ssRNA	Positive-sense single-stranded RNA
SVIR	Susceptible–Vaccinated–Infected–Removed
WHO	World Health Organization
US	United States
UK	United Kingdom
